# Sex Disparities of the Sacroiliac Joint: Focus on Joint Anatomy and Imaging Appearance

**DOI:** 10.3390/diagnostics13040642

**Published:** 2023-02-09

**Authors:** Sevtap Tugce Ulas, Torsten Diekhoff, Katharina Ziegeler

**Affiliations:** 1 Department of Radiology, Charité–Universitätsmedizin Berlin, Campus Mitte, Humboldt–Universität zu Berlin, Freie Universität Berlin, 10117 Berlin, Germany; 2 Berlin Institute of Health, Charité–Universitätsmedizin Berlin, 10117 Berlin, Germany

**Keywords:** sacroiliac joint, sex disparities, anatomical variation, axial spondyloarthritis, osteitis condensans

## Abstract

The sacroiliac joint (SIJ) is an anatomically complex joint which, as a functional unit with the pelvis and spine, is of decisive biomechanical importance for the human body. It is also a commonly overlooked source of lower back pain. Like the entire bony pelvis, the SIJ exhibits major sexual dimorphisms; thus, the sex-dependent evaluation of this joint is becoming increasingly important in clinical practice, both anatomically with joint shape variations and biomechanical differences as well as in terms of image appearance. The influence of the SIJ shape, which differs in women and men, is crucial for the different biomechanical joint properties. These differences are important in the development of joint diseases at the SIJ, which shows a specific difference between the sexes. This article aims to provide an overview of sex disparities of the SIJ regarding different anatomical and imaging appearances to further understand the insights into the interplay of sex differences and SIJ disease.

## 1. Introduction 

The sacroiliac joint (SIJ) is the articulation surface between the sacrum and the ilium and plays an important role in the distribution of axial load between the spine and pelvis [1]. It provides stability and offsets the load of the trunk to the lower limbs and is, therefore, one of the important mechanical axes of the human body [1,2]. The complex structure of the ligaments of the SIJ is of great importance for SIJ stability [3]. As such, it is estimated to be the source of lower back pain in up to 30% of cases [4]. In women, lower back pain is more commonly associated with degenerative or mechanically induced diseases of the SIJ [5]. Various factors play a role in this: Anatomical differences in the configuration of the SIJ have recently received increased scientific focus, as they are associated with a higher probability of degenerative changes [6]. In addition, major sex-specific differences in the frequency of anatomical variations in the sacroiliac joint have been reported [6,7].

This understanding plays an essential role in the differentiation of degenerative or inflammatory diseases and pathological findings of the SIJ and makes knowledge of the functional anatomy and biomechanical aspects of the SIJ particularly significant [2,8]. In this regard, sex differences are not only relevant in the anatomy of the sacroiliac joint but are also associated with differences in the expression and clinical presentation of diseases of the SIJ [9,10]. In addition, the enormous technological advances developed in recent years allow a more sophisticated investigation of the human body and its anatomical and pathophysiological significance in the clinical presentation of SIJ diseases [11]. In this context, the application of microscopic [12], molecular, genetic, and now radiological and functional imaging techniques [13] have become increasingly important in the evaluation of the SIJ. The development of this virtue of forefront methods allows in this way a much deeper understanding of different SIJ diseases and thus can provide a significant contribution to therapeutic strategies. 

The aim of this article is to give an overview of sex-specific differences of the SIJ regarding anatomical and imaging appearances to further understand the interplay of sex disparities and SIJ disease.

## 2. Anatomy of the SIJ

The anatomy of the sacroiliac joint is characterized by the complexity of its structure as well as the resulting biomechanical significance [2]. It provides an articular connection between the ilium and sacrum [2], which is braced by several ligaments and muscularly via the piriformis and gluteus maximus muscle.

The mechanical load is distributed to the ligamentous structures, muscles, and fascia. In addition to the stability function and the transmission of force from the legs to the body, it allows only limited mobility in the sense of a tilt-like movement of the sacrum against the pelvic rings, also known as nutation (forward and downward movement) and counternutation (backward and upward movement) [2,14]. Furthermore, sideway movements of the SIJ are possible, but only with a very limited range. This is due to the tightness of the well-developed fibrous apparatus, which straddles adjacent and non-adjacent vertebrae and the sacrum, as well as the specific anatomy of the SIJ [2]. The sacrum is characterized by its wedge shape and the many small grooves and ridges on its joint surface [15,16]. These make a decisive contribution to friction through the surface structure in the sense of frictional stability under axial force [15].

### 2.1. Biomechanical Relevance of the SIJ

The SIJ plays an important role in the mechanical stability of the human body due to its anatomical and biophysical properties [2,17]. In this context, the main function of the joint biomechanically is to transmit forces from the legs to the body, which is crucial, especially in bipedal walking [18,19]. This results in the SIJ being subjected to high mechanical strain.

This biomechanical stress has a significant influence on the induction of inflammatory processes (so-called “mechanoflammation”) in the SIJ [20,21], which are of particular importance in the development of various inflammatory conditions of the SIJ [22]. Similarly, the SIJ is subject to intense mechanical stress during pregnancy and childbirth [9], as the movement of the sacroiliac joint regulates the width of the pelvis; further strain is contributed by the increased weight and shift in the center of gravity during late pregnancy. The influence of the joint shape is decisive for the distribution of the mechanical forces over the entire joint [17].

### 2.2. Variation of the SIJ Anatomy

**Figure 1:**
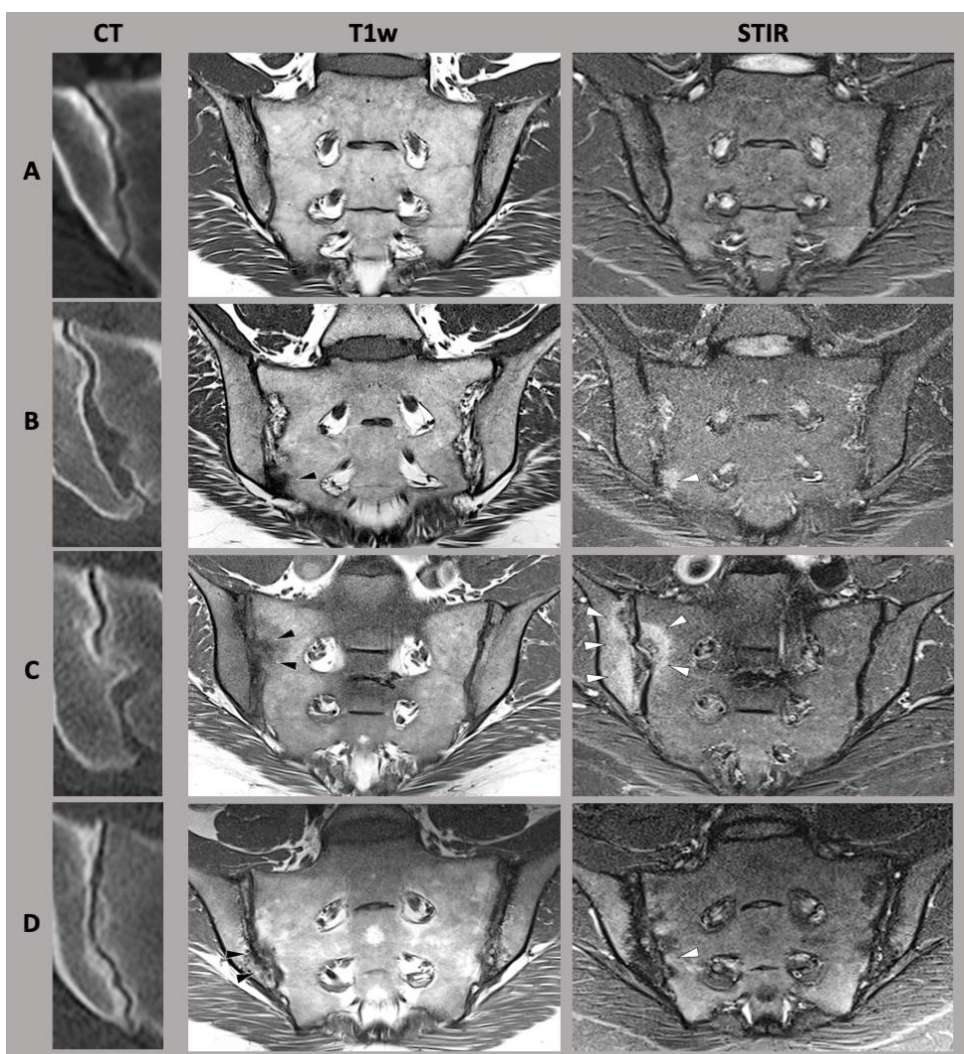
A selection of sacroiliac joint form variation. (CT) computed tomography, (T1w) T1-weighted magnetic resonance imaging sequence, (STIR) short-tau inversion recovery magnetic resonance imaging sequence. (**A**) typical sacroiliac joint form, (**B**) accessory joint facet, (**C**) sacroiliac complex, (**D**) crescent-shaped ilium. Adapted with permission from Refs. [6,24]. Copyright 2021-22, copyright Katharina Ziegeler.

Initial attempts at differentiation of joint shapes have been evaluated in detail by Prassopoulos et al. [23]. In their work, they distinguished six different shape variants of the SIJ as follows: the accessory joint facet, typically located dorso-inferiorly to the main joint; the sacroiliac complex, which constitutes a convex bulge of the ilium, corresponding to a concave sacral facet; the bipartite iliac bone plate, which may look like a slim bony defect parallel to the joint in its dorsal part; the crescent-shaped ilium, which leads to an overall more saccular shape of the sacroiliac joint; semi-circular notches (also referred to as “defects”) on both iliac and sacral side of the joint; and sacral ossifications center, typically located at the ventral portion of the joint [23] (also see Figure 1).

Of particular importance are shaped variants associated with altered biomechanics [8]. Initial studies suggest that these have some association with chronic degenerative changes of the SIJ, possibly due to increased mechanical stress or altered relative movement and therefore differences in load distribution [6]. In addition, shape variants are also associated with an increased likelihood of the occurrence of inflammatory changes at the SIJ. SIJ form variation is especially common in patients with lower back pain along with symptomatic joint disease [7,24].

However, the classification of the joint form is rather inconsistent and variable reproducibility is reported in the literature [24,25]. Furthermore, data on the specific characteristics of the joint forms of the disease process are still missing.

### 2.3. Sex-Specific Differences in Anatomy and Biomechanics

**Figure 2:**
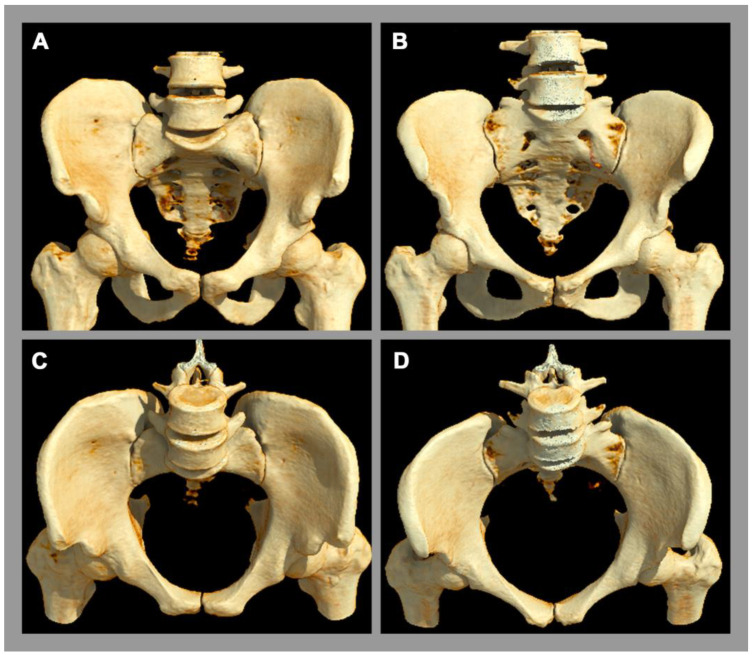
Anatomy of the pelvis. A 3D computed tomography reconstruction. (**A**,**C**) Male pelvis. (**B**,**D**) Female pelvis. There is obvious sexual diamorphism in the pelvic anatomy. The male pelvic cavity is longer and more conically configurated with a longer and narrower sacrum, while the female pelvic cavity is narrower and cylindrically orientated.

There is a well-established sexual dimorphism of the SIJ with consecutively more degenerative or mechanical stress-induced disease in the female SIJ [2]. In women, the ligaments of the SIJ are more flexible and the center of gravity is more ventrally located [8]. In the male pelvis, especially the inter-cristal measurement is larger than in the female pelvis [2]. The articular facet at the base of the sacrum for the 5th lumbar vertebra occupies more than one-third of the width of the base of the sacrum in males [2]. In females, this corresponds to less than one-third, whose sacrum is relatively wider [2]. In addition, the pelvic cavity is longer and more conically configured in males, whereas the female pelvic cavity is shorter and more cylindrical orientated [2], see Figure 2.

The surface area of the sacroiliac joint is slightly larger in men than in women [26] with a typically thinner sacrocartilaginous alignment, suggesting a higher tolerance towards biomechanical load in men [2,16]. The SIJ in women is characterized by higher SIJ mobility [8], which is primarily enabled by a lower curvature of the SIJ surface in women. Correspondingly, these differences in sacroiliac joint anatomy lead to differences in biomechanical and biophysical properties with higher rates of SIJ misalignments in women [27]. This is also associated with an increased rate of lower back pain in female patients [28]. 

Previous studies of our group showed that the “typical” joint shape is seen preferentially in men (85.9% vs. 37.9% in women) [6]. Consecutively, shape variants of the SIJ are significantly more common in women [6]. The bipartite iliac bone plate is most frequently found in women (21.9% vs. 0.7% in men), followed by the accessory joint facet (12.7% vs. 4.1% in men) [6].

These findings are of particular importance when investigating the relationship between joint shape and disease development in the sacroiliac joint since degenerative or inflammatory SIJ changes are more frequently found in anatomically atypical joints [24]. 

## 3. Imaging of the SIJ

The sacroiliac joint is usually not in the focus of imaging when lower back pain is investigated. Thus, indications for a dedicated assessment are limited. It is regularly performed in the first assessment of patients with inflammatory lower back pain and suspected axial spondyloarthritis (axSpA), where imaging is the single most predictive method to objectify current active or preceding inflammation [29,30,31,32]. In axSpA, imaging of the SIJ plays a crucial role, in concert with clinical and laboratory parameters, in both primary diagnostic assessment and therapy monitoring [33]. The focus of the initial presentation is on the differentiation between inflammatory and degenerative changes of the SIJ. Thereafter, imaging is used to monitor active inflammation and structural damage in therapy studies or to depict complications from long-standing diseases. In addition, imaging of the SIJ is performed after traumatic events to evaluate fractures of the sacrum, and when osteoporotic insufficiency lesions are suspected. Nonetheless, the SIJ is co-imaged on many examinations, even if no specific pathology is sought on the SIJ itself. For example, it is included in an X-ray or magnetic resonance imaging (MRI) of the pelvis or lumbar spine, as well as in CT examinations of the abdomen and pelvis in the context of assessing inflammatory bowel disease, staging for neoplastic diseases and others, which are performed more frequently in clinical routine.

Various imaging modalities are available for the assessment of the SIJ. The choice of the right imaging method depends on whether the focus is on inflammatory or morphological structural changes [34].

### 3.1. X-Ray

An X-ray of the SIJ usually plays a role in daily routine as initial imaging to assess structural changes in the context of axSpA. It is inexpensive, available in most institutions, and can be performed quickly. The ASAS handbook recommends a standard anterior-posterior (a.p.) radiograph of the pelvis, which gives a nice overview of the anatomy and also allows the assessment of the hips. Radiography of the SIJ is focused on this area and, therefore, exhibits minimal radiation but does not depict other joints for differential diagnoses. Imaging using the so-called Ferguson technique, in which the X-rays are projected from caudally with an inclination of 30–35° to the middle portion of the pelvis, allows direct imaging of the SIJ, in which the articular surface of the lower part of the SIJ, in particular, can be better assessed [35]. However, this advantage is bought by an increase in ionizing radiation. Some institutions perform other projections in addition—for example, an inlet and outlet view of the pelvis and 15° laterally angulated views for each SIJ—to increase the sensitivity for pathologies. However, in general, such an approach is discouraged, as the overall diagnostic accuracy of X-ray is comparably low and only marginally increases with additional projections. A lateral image of the sacrum is usually only necessary in cases of suspected fractures.

The X-ray allows only the visualization of structural bone changes, such as sclerosis, erosions, and ankylosis, and can, thus, provide initial indications for the differentiation from joint diseases occurring in the context of degenerative changes, such as osteitis condensans. Nevertheless, the X-ray of the SIJ remains crucial for qualitative differentiation between radiographic (r-axSpA) and non-radiographic axSpA (nr-axSpA) according to the modified New York or ASAS criteria [36]. Studies showed that female axSpA patients had less r-axSpA according to the modified New York criteria than men [37], and both structural damage and radiological progression are more common in male axSpA patients [9]. Women show a longer diagnostic delay; one reason is that typical radiographic changes appear later in the course of the disease [10].

Due to the complexity of the SIJ anatomy and its orientation, X-rays alone are often unsatisfactory for the depiction of joint form variations and smaller pathologies, and therefore three-dimensional imaging methods are usually necessary for further evaluation of the SIJ and its anatomy. For this reason, the classification of variations of the SIJ anatomy in X-ray has not yet been sufficiently investigated.

### 3.2. MRI

In contrast to X-ray, MRI allows three-dimensional visualization of SIJ and a detailed assessment of structural lesions and active inflammation. In addition to the bony changes described above, it also allows the depiction of possible changes in the periarticular soft tissues, such as the joint capsule and entheses, and, most importantly, the bone marrow. In routine clinical practice, an MRI of the SIJ is used primarily for visualization of acute inflammatory changes in the context of axSpA, which is particularly crucial in the early stage of this disease. After the publishing of the ASAS classification criteria, bone marrow edema has played a central role in diagnosing axSpA. However, it can also occur in the context of biomechanical processes.

Precise and sensitive imaging of the SIJ in clinical routine as well as in clinical studies can be achieved with a small number of sequences. The aim is to distinguish between inflammatory and structural or degenerative changes in the pathological findings. A T1-weighted sequence allows indirect visualization of the bone and is used to assess structural changes such as sclerosis, erosions, fat metaplasia, and ankylosis. Fat metaplasia appears hyperintense in T1-weighted images. Inflammatory changes such as bone marrow edema or enthesitis can be visualized with either the fat-saturated T2-weighted sequence or the short-tau inversion recovery sequence (STIR). Here, these inflammatory changes are hyperintense. In most cases, an examination without the application of a contrast agent is sufficient for this purpose, as both the STIR and the T1-weighted sequence with the contrast agent adequately depict the most relevant inflammatory changes in the bone marrow [38]. Both sequences are performed in the paracoronary plane (angled parallel to the axis of the second sacral vertebra in the sagittal plane). While both sequences are usually sufficient to monitor the disease when the diagnosis is established, for primary diagnostic purposes two additional acquisitions are recommended: a paraxial STIR and an erosion-specific sequence. The oblique-axial STIR is used to better localize inflammatory lesions in the SIJ and assess whether they are restricted to the mechanical load zone. For the erosion-specific sequence, a high-resolution 3D-gradient echo sequence is nowadays standard [39] but also other modern sequences have been proposed [40,41]. These sequences take more and more early diagnoses into account, where structural lesions can be subtle and are less well depicted in standard T1.

Sex differences in the evaluation of MRI images must also be considered. Since more biomechanical forces have an effect on the female SIJ, among other things due to their specific anatomy, these changes are correspondingly more frequent, which can be associated with both degenerative and inflammatory changes. Thus, bone marrow edema on MRI is more common in women than in men without axSpA, which is another reason for the difficulty in diagnosing axSpA. In addition, studies showed that form variants of the SIJ occur more frequently in women [7,42], which can be visualized in the T1-weighted sequence. Since form variations are also associated with changes in the biomechanical property, structural or inflammatory changes of the bone related to form variations can also be visualized on MRI. However, MRI might not be the most sensitive and specific modality for an accurate assessment of the anatomy and possible shape variations, because it is limited by its spatial resolution which is inferior compared to CT (3–5 mm vs <1 mm through-plane resolution) and the limited coverage of the image stack.

### 3.3. CT

Due to its high spatial resolution, CT allows a detailed assessment of the complex SIJ anatomy and, for this reason, is also the reference standard for direct bone imaging. This allows excellent visualization of structural changes, such as erosions, sclerosis, and new bone formations. In clinical practice, CT of the SIJ has tended to be performed only in a few number of cases, when MRI is not available or image assessment is inconclusive. However, new methods for reducing the radiation exposure to the level of radiography or below allow for a broader indication for CT in patients with an early disease or unclear MRI findings.

CT has the widest clinical application, especially in the context of fracture evaluation of the SIJ. It can be performed quickly in the acute clinical setting and is superior to conventional radiography, as it allows superior multidimensional imaging of the anatomical structures. Thus, it also serves as a reference standard in the classification of anatomical variants of the SIJ [43,44,45]. Conventional CT does not allow visualization of bone marrow edema. With recent techniques and further developments in CT imaging, more sophisticated possibilities in the more accurate evaluation of pathologies at the SIJ are feasible. 

### 3.4. New Imaging Modalities

Technological developments have enabled a broader application of new imaging modalities in SIJ imaging. Although reference standard in bone imaging, the use of CT has been rather limited in routine clinical practice. One of the main reasons is the comparably high radiation exposure. New developments and the use of low-dose CT protocols can address this and allow a broader application in clinical practice. In addition, CT techniques that allow material-specific characterization in low- and high-energy spectra, such as dual-energy CT or photon-counting CT, allow the visualization of bone marrow edema using so-called virtual non-calcium images. While this technique is, today, not possible with low-dose acquisitions, it shows a promising development in the assessment of bone marrow lesions and active inflammation with modern versions of CT.

In contrast to CT, MRI shows weaknesses in the evaluation of structural changes. Often, these lesions are overestimated in MRI because the T1-weighted sequence used for this purpose only allows indirect bone imaging. For this reason, the assessment of new MRI sequences is of particular importance. One possibility is to synthesize CT-like images using artificial intelligence algorithms [41,46,47]. Another method is the direct use of magnetic properties of the bone for its direct visualization with the so-called susceptibility-weighted imaging sequence (SWI) [40,48,49] or the use of new pulse sequences with ultra-short echo times (UTE) or zero echo time (ZTE) [50,51,52], which use the more precise representation of the water content for the bone imaging. Tissue characterization can also be performed with the latest MRI techniques using T2-weighted mapping sequences, which have already been investigated in the first few studies at SIJ [53] and may contribute to the differentiation of diseases and the understanding of pathophysiological processes.

However, all these modalities are still the subject of ongoing research and are not used in clinical practice. 

## 4. Diseases of the SIJ

The high mechanical load acting on the SIJ is a possible approach to explaining the frequent occurrence of diseases at the SIJ. A distinction must be made between degenerative and mechanical-stress-induced diseases such as osteoarthritis and osteitis condensans and inflammatory diseases of the sacroiliac joint, which include sacroiliitis and axial spondyloarthritis. Often, these diseases are difficult to distinguish based on clinical phenotype alone [9], making imaging essential for diagnosis.

### 4.1. Sex-Specific Differences in Imaging Appearance of Mechanical or Degenerative SIJ Diseases

**Figure 3:**
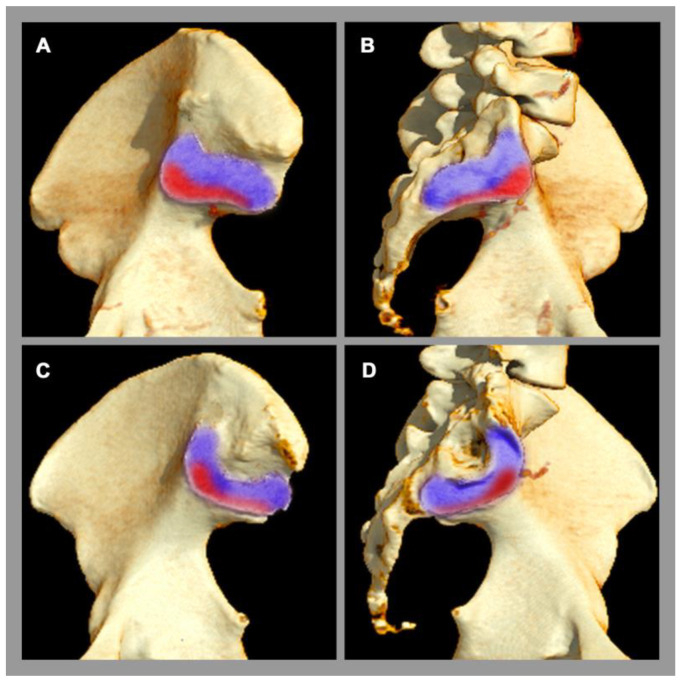
Sacroiliac joint load zones. 3D computed tomography reconstruction. (**A**,**C**) Lateral view of the ilium. (**B**,**D**) Lateral view of the sacrum. Male pelvis (**A**,**B**). Female pelvis (**C**,**D**). There are sex differences in the distribution of the load zone (red area) with respect to the rest of the joint surface (purple area). In women, the center of gravity is more ventrally located.

Osteitis condensans ilii is a frequent pathology of the SIJ [5], which is classified as a non-inflammatory disease. Its origin is attributed to mechanical stress and imbalance across the SIJ, e.g., during pregnancy and childbirth [2], causing a chronic stress response. Clinically, affected patients present with chronic back pain, which typically begins before the age of 45 [5]. Osteitis condensans ilii is significantly more common in women than in men, possibly due to the different biomechanical properties of the SIJ and flexible ligamentous structures in the female pelvis [5], especially concerning delivery. The typical imaging finding at the sacroiliac joint is sclerosis and its specific distribution pattern in the anterior and inferior part of the SIJ with a typical triangular shape (also called “hyperostosis triangularis ilii”) [5]. In addition, bone marrow edema can also be seen in osteitis condensans ilii, but this is not specific, since other factors, such as sporting activities, can also lead to bone marrow edema in the SIJ in the region of mechanical stress [54,55], see Figure 3.

In the diagnostic process of degenerative conditions of the SIJ, naturally occurring aging should always be considered. These imaging lesions are to be regarded as a consequence of repetitive, inappropriate biomechanical stress, which is accompanied by joint wear and may result in lower back pain. Besides patchy small-sized focal spots of bone marrow edema, sclerosis, and osteophytes are typical findings, but their distribution pattern in the general population differ between the sexes: In men, the degenerative changes are mainly localized in the ventral part of the SIJ, whereas in women they are more likely to be found ventrally and dorsally [6,56]. In addition, a different age distribution is also evident, where degenerative changes in women show an early peak between 45 and 54 years of age, whereas, in men, a steady increase in degenerative lesions of SIJ with increasing age has been shown [6]. Furthermore, SIJ degeneration in imaging is associated with degenerative changes of the lumbar spine in men but not in women [57].

Anatomical variants are strongly associated with degenerative or mechanical conditions of the SIJ (women 92.7% and men 55.0%) [7]. The impact of the SIJ form variation on the biomechanical property also shows corresponding clusters of degenerative changes [7].

### 4.2. Sex-Specific Differences in Imaging Appearance of Inflammatory SIJ Diseases

AxSpA is a chronic inflammatory disease that primarily affects the SIJ and has historically been considered primarily in male patients [58]. While newer studies suggested a more equal gender distribution in early axSpA [9,10,59], these results might, in part, be attributable to difficulties in interpreting MR imaging data in women and an over-emphasis of bone marrow edema [60]. Early diagnosis is essential for further disease progression and improvement in quality of life. Imaging characteristics of axSpA are sclerosis, erosion, bone marrow edema, fat metaplasia, and ankylosis [33,61].

Sex differences play a crucial role in the clinical presentation of axSpA [9]. Structural damage, such as ankylosis, is more common in men, while peripheral manifestations are significantly more common in women, with a higher risk of pain and fatigue [9,62,63]. In addition, women show higher disease activity and lower response to therapy than men [64]. Bone marrow edema is considered an important imaging finding according to ASAS MRI group recommendations [65]. However, bone marrow edema has limited specificity, especially in women, because many other causes can also lead to bone marrow edema [54,55], such as pregnancy and delivery [66]. These might be reasons for the longer diagnostic delay in female axSpA patients [10].

In a recent study, we demonstrated that the diagnostic performance of established imaging markers on MRI is significantly lower in women than in men [67]. In particular, this is the case for ankylosis, which is evaluated as a hallmark in axSpA, which provides the risk of false-positive findings in women [67]. In addition, sex-specific distribution patterns of sclerosis and fat metaplasia in imaging appearance of the SIJ could be shown with more sclerosis in females, while fat metaplasia was more common in males [67].

Furthermore, men with axSpA were more likely to have atypical joint forms (32.2% vs. 13.9%) [7]. A recent study also showed the association of several SIJ form variations with erosions and bone marrow edema, which are hallmarks of axSpA [24].

## 5. Clinical Impact of Sex Differences in the SIJ

It is essential to understand the sex-dependent differences in the biomechanical properties of the SIJ when assessing and evaluating imaging findings. Through them, an important differentiation of the various disease entities can be made considering the influences of sex differences. In this way, misclassifications and false diagnoses can be avoided. On the other hand, knowledge of sex differences in SIJ anatomy provides important information for a better understanding of the pathophysiological background of e.g., inflammatory SIJ diseases, since such diseases are associated with altered or non-physiological force distribution and can, thus, induce or maintain inflammatory changes at the joint.

In addition to these changes in anatomical structure, including differences in biomechanical load, sex differences in clinical presentation are also delineable. For example, in axSpA, women are known to report more pain, stiffness, and fatigue, and activity impairment is more common than in men [9]. In addition, female axSpA patients are more commonly afflicted by peripheral manifestations of axSpA with more tender/swollen joints and enthesitis [9]. Furthermore, studies showed significantly higher disease activity and lower response to therapy in women compared to men [9,64]. More frequently, women with widespread pain are also misdiagnosed as having fibromyalgia or have a more concomitant diagnosis of depression [9]. A differentiated view is, therefore, of crucial importance, especially in early diagnosis. All of these factors result in the fact that early and correct diagnosis of inflammatory lower back pain in women remains a challenge. This requires appropriate training and awareness of rheumatologic and non-rheumatologic health professionals on the sex-specific clinical and radiological manifestations of axSpA.

For this reason, sex-dependent data collection is of great importance to better understand these processes. On the basis of these findings, sex-specific diagnostic and classification criteria might be developed, leading to an overall improvement in diagnostic accuracy.

## 6. Conclusions

In conclusion, significant differences in the morphology and biomechanics of male and female SIJ exist, which are associated with different patterns of SIJ findings. In women, joint form variations are significantly more common, with a resulting more diverse degenerative pattern that requires special consideration in the image interpretation in suspected osteoarthritis and potentially in the development of classification and of diagnostic criteria. In axSpA, marked sex differences in the diagnostic performance of commonly used imaging features of sacroiliitis have been observed—possibly also owing to the above-detailed differences in biomechanics and anatomy. However, this narrative review only aims to outline the current scientific knowledge on the topic, which to date is limited to mainly in-silico and retrospective clinical studies. We hope to encourage further studies with prospectively enrolled patients to implement the latest technological developments and to investigate the understanding of the clinical impact, hopefully leading to specifically tailored imaging for both sexes.

## Data Availability

The data presented in this study are available on request from the corresponding author.
